# Parasitic Appendicitis From Past to Present in Turkey

**Published:** 2010-09

**Authors:** O Engin, S Calik, B Calik, M Yildirim, G Coskun

**Affiliations:** 1 Izmir Bozyaka Training and Research Hospital,Surgery Department, Izmir-Turkey; 2 Department of Clinical Microbiology, Urla State Hospital, Izmir-Turkey; 3 Buca Seyfi Demirsoy Large State Hospital, Surgery Department,Izmir-Turkey; 4 Department of Surgery, Izmir Bozyaka Training and Research Hospital, Izmir-Turkey; 5 Department of Pathology Buca Seyfi Demirsoy Large State Hospital, Izmir-Turkey

**Keywords:** Parasitic Appendicitis, *Enterobius vermicularis*, Acute Abdomen, *Taenia*

## Abstract

**Background:**

Understanding the etiology of appendicitis is important for developing effective treatments the relationship between parasitic appendicitis and various socio-cultural factors were examined, particularly with respect to the incidence of literacy. The aim of the article was to research the relations between parasitic appendicitis and literacy ratio in population.

**Methods:**

Cases of parasitic appendicitis resulting in surgery performed at Buca Seyfi Demirsoy Large State Hospital Surgery Clinic between 2002 and 2009 were retrospectively reviewed and classified according to age, sex, type of parasite, morbidity, and mortality. Studies conducted in different regions of Turkey as well as in other countries were reviewed to determine if there was a relationship between parasitic appendicitis and literacy.

**Results:**

Of the 1,969 appendectomy cases reviewed, nine were classified as parasitic appendicitis (0.45%). *Enterobius vermicularis* was observed in seven cases and *Taenia* spp. in two. The average age was 26.4 yr. No morbidity or mortality was found.

**Conclusion:**

The data were compared with a retrospective review of studies conducted in the same regions and a decrease in the rate of parasitic appendicitis was observed during the period between the two reviews. It was determined that a low literacy rate was associated with an increase in the incidence of parasitic appendicitis. Observations made between different countries also produced similar results. In countries where the incidence of parasitic appendiciticis was greater than 1.5%, the literacy rate was less than 88%. To avoid appendectomy resulting from parasites, it is important to increase education and literacy. In some areas, individuals with appendicitis undergo surgery due to a lack of education or poor literacy.

## Introduction

The relationship between the base of the appendix and the cecum does not change, but the terminal end of the appendix can be located at different positions. The combination of the three tenia of the colon forms the longitudinal muscles of the appendix, which lead to the location of the appendix. The positions related to the point of the terminal end of the appendix can be identified as retrocaecal, pelvic, subcaecal, ileocaecal and right pericolic (1, 2).

Acute appendicitis occurs in almost 7% of the population. It is the most common, emergent surgical disease, and its main cause is the obliteration of the lumen of the appendix. Fecaliths, lymphoid hyperplasia, parasites, seeds of vegetables and fruits, barium enemas, and tumors in the large intestine and appendix, are all etiologies of acute appendicitis (1, 3–5).

In this study, cases of appendicitis due to parasites were examined and compared to cases in other studies conducted in the same regions over a number of years. Then, studies conducted in different parts of Turkey were compared with literacy rates in those regions, while a comparison was also made between various countries, and the relationship between rates of parasitic appendicitis and literacy was explored by analyzing the different rates.

## Materials and Methods

Specimens with parasites were taken from patients who underwent appendectomy for appendicitis, and the specimens obtained were then placed in sterilized containers with 10% normal saline solution and sent to the pathology laboratory. Age, gender, type of parasite, morbidity and mortality were retrospectively analyzed.

Results from studies conducted in the same region were then compared to the results of this study, while various studies conducted in different regions of Turkey and other countries were examined to determine whether a relationship between parasitic appendicitis and literacy exists. Finally, we examined ways to protect individuals from parasitic appendicitis.

## Results

**Fig. 1:**
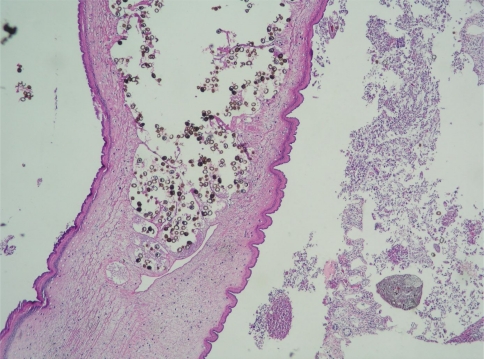
The case in our series. *Taenia* spp. H&E x 4

**Fig. 2:**
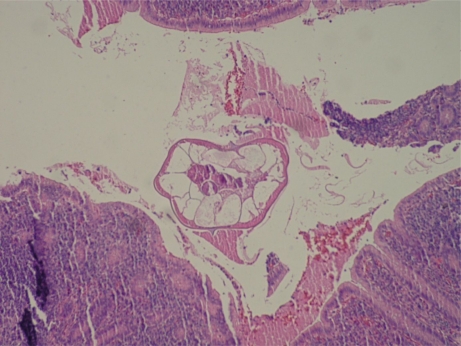
The case in our series. *Enterobius vermicularis* H&E x 2

The number of cases of parasitic appendicitis among 1,969 cases of appendicitis observed was studied in Izmir, Turkey, between 2002 and 2009. The number of cases of parasitic appendicitis was nine (0.45%). *Taenia* spp. (Fig. 1) was observed in two cases, and *Enterobius vermicularis* (Fig. 2) was found in seven cases. The female to male ratio was 8:1, and the average age was 26.4 yr. The incidence among all cases was 0.45%. The main complaint was abdominal pain, and on abdominal examination, symptoms were guarding and rebound in the right lower quadrant. Patients who underwent surgery because of a diagnosis of acute appendicitis were recovered and discharged from the hospital without complications. On histopathologic examination, fecaliths and parasites were observed in two cases, but in seven cases, parasites were seen in the lumen of the appendix.

## Discussion

Appendicitis was more common in men than in women, despite a male to female ratio of 1.4:1. It is expected that 8.6% of males and 6.7% of females will develop acute appendicitis in their lifetime. Young age is a risk factor, since nearly 70% of cases were under 30 years of age. The appendix can be infiltrated by many materials, such as fecal material, microbes, and parasites, and because most of these materials are found in the intestinal lumen, they can enter the lumen of the appendix, although they may not necessarily result in symptoms. However, they can cause inflammation at any time, and thus, appendicitis. If parasite ova or cysts block the lumen, or are lodged in the lumen, inflammation of the appendix may develop. The typical clinical process begins with intermittent stomach ache-like cramps, which are caused by the blockage of the lumen of the appendix. Pain can be present partially or exclusively around the umbilicus, and can be difficult to localize. Typically, the ache-like cramps are followed by nausea, although this may not necessarily occur. When inflammation becomes transmural and causes pyogenesis of the peritoneum covering the right lower quadrant, it is typically located in the right lower quadrant. With a change in the degree of pain, the obtuse colic pain is replaced by constant and severe pain (6–8).

The parasites identified in our study were *E. vermicularis* and *Taenia* spp. *Enterobius vermicularis*, a nematode, is a parasite mostly located in the cecum. It can lead to the development of appendicitis, and is widely found in children throughout the world. Pregnant female parasites do not lay eggs in the intestines, but move from the intestinal wall to the intestinal cavity, before laying eggs around the anus. However, the female moves from the anus at night and dies after laying its eggs in the perianal site. The eggs can be spread because of scratching the irritation caused by the helminth parasite. Eggs can also be absorbed during digestion, or can be borne by airborne dust, which can be inhaled through the mouth or swallowed with food. The mature helminth emerges during the transition through the jejunum and ileum, and is located in the cecum. The transmission of parasites to humans can occur in various ways. Occasionally, larvae hatch and develop in the perianal site and enter the intestines, resulting in retroinfection (9–12).

In a study carried out in Izmir-Turkey between 1990 and 1996, it was observed that there were 45 parasitic appendicitis in 2473 cases having primary appendicitis. The rate of parasitic appendicitis was 1.8% (13).

Our study was also made in the same region, but so many years after the previous study; that was between 2002 and 2009. Number of cases having parasitic appendicitis was 9, and the rate of incidence was 0.45%. According to the data of 2000, the rate of literacy in Izmir was 96.26% in men and 87.41% in women (13).

Literacy rates in Turkey were collected from a review of 2,000 general census forms and from the website of the Ministry of Education (17) (Table 1). We also included other countries in this study. Literacy rates for the above countries were obtained from data from the United Nations, with the exception of Japanese data, which was obtained from the CIA world database (23, 24). Data comparing the various countries in this study are presented in Table 2.

**Table 1:** Data obtained from cities in Turkey

**City (Reference)**	**Parasitic app ratio (%)**	**Male literacy ratio (%)**	**Female literacy ratio (%)**
Izmir (13)	0.45	96.26	87.41
Ankara (15)	0.3	97.14	89.34
Eskisehir (16)	1.44	96.84	89.11
Adana (14)	4.8	93.64	80.26

**Table 2:** Data obtained from countries included in this study

**Country**	**Parasitic app ratio (%)**	**Literacy total ratio (%)**	**Literacy male ratio (%0)**	**Literacy female ratio (%)**
Japan (20)	0.32	99	99	99
Brazil (21)	1.5	87.3	87.4	87.2
Iran (18)	2.9	77.1	83.8	77.1
Kuwait (22)	5.6	82.4	84.3	80.3
South Africa (19)	5.62	85.6	86.3	85
Malaysia (5)	16	87.9	91.7	84.0

These data show that, as the literacy rate in regions of Turkey decreases, the rate of parasitic appendicitis increases. This connection is observed in countries around the world. In the countries included in this study where the literacy rate was<88%, the incidence of parasitic appendicitis was ≤1.5%.

Parasitic infections rarely cause acute appendicitis. To avoid parasitic infestation, individual education and standards of public health and hygiene need to be raised; contaminated water, food and clothing should be avoided; and the number of modern and hygienic restaurants should be increased. Moreover, emphasis should be placed on preventive and therapeutic approaches. If parasitic infestations are prevented, the rate of appendicitis caused by parasitic appendicitis will decrease (7, 25).
